# Biomacromolecules and Bio-Sourced Products for the Design of Flame Retarded Fabrics: Current State of the Art and Future Perspectives

**DOI:** 10.3390/molecules24203774

**Published:** 2019-10-20

**Authors:** Giulio Malucelli

**Affiliations:** Department of Applied Science and Technology, and local INSTM Unit, Viale Teresa Michel 5, 15121 Alessandria, Italy; giulio.malucelli@polito.it; Tel.: +39-0131-229369

**Keywords:** flame retardance, cotton, jute, polyester, silk, wool, biomacromolecules, caseins, whey proteins, hydrophobins, chitosan, deoxyribonucleic acid, phytic acid, tannins, layer by layer technique, cone calorimetry, flammability tests

## Abstract

The search for possible alternatives to traditional flame retardants (FRs) is pushing the academic and industrial communities towards the design of new products that exhibit low environmental impact and toxicity, notwithstanding high performances, when put in contact with a flame or exposed to an irradiative heat flux. In this context, in the last five to ten years, the suitability and effectiveness of some biomacromolecules and bio-sourced products with a specific chemical structure and composition as effective flame retardants for natural or synthetic textiles has been thoroughly explored at the lab-scale level. In particular, different proteins (such as whey proteins, caseins, and hydrophobins), nucleic acids and extracts from natural sources, even wastes and crops, have been selected and exploited for designing flame retardant finishing treatments for several fibers and fabrics. It was found that these biomacromolecules and bio-sourced products, which usually bear key elements (i.e., nitrogen, phosphorus, and sulphur) can be easily applied to textiles using standard impregnation/exhaustion methods or even the layer-by-layer technique; moreover, these “green” products are mostly responsible for the formation of a stable protective char (i.e., a carbonaceous residue), as a result of the exposure of the textile substrate to a heat flux or a flame. This review is aimed at summarizing the development and the recent progress concerning the utilization of biomacromolecules/bio-sourced products as effective flame retardants for different textile materials. Furthermore, the existing drawbacks and limitations of the proposed finishing approaches as well as some possible further advances will be considered.

## 1. Introduction

When exposed to the action of a flame or a heat flux, most textile materials easily ignite and burn: this behavior severely limits their utilization in several application fields, where fire resistance is mandatory. In this context, from the 1950s onwards, specific additives, i.e., embedded of surface-treated flame retardants (FRs) able to slow down the flame propagation and even to prevent the burning of materials have been designed and produced [1,2,3,4]. Specifically concerning fibers and fabrics, several classes of flame retardants have been developed to date, differing in chemical structure and composition, as well as the flame retardant mechanism involved [5,6,7,8]. The development of FR finishing systems for fibers and fabrics has exhibited continuous progress, especially in the last 15–20 years, during which academic and industrial research has been focused on conceiving, synthesizing and utilizing large-scale durable treatments, either for natural (mostly cellulosic) or synthetic textile substrates [9,10,11,12,13,14,15,16]. Indeed, effectiveness, durability (i.e., resistance to environmental conditions), cost and comfort issues were the main targets of the performed research. Therefore, halogenated organic (mainly brominated and chlorinated), phosphorous and/or nitrogen-based and inorganic flame retardants have been fruitfully employed for providing fire resistance to different types of textiles, either natural or synthetic, as well as to their blends (such as cotton-polyester blends) [17,18]. Nonetheless, despite their effectiveness, some halogen-based products, such as polychlorinated biphenyls, decabromodiphenyl or pentabromodiphenyl ethers were recently banned by USA and EU communities, owing to their high toxicity for animals and human beings [19,20].

For all these reasons, this research addressed the design of effective halogen-free FRs, in particular on phosphorus- or phosphorus/nitrogen-based compounds [21]: in doing so, several flame retardant systems were proposed; some of them, specifically suitable for cellulosic substrates (namely N-methylol phosphonopropionamide derivatives (Pyrovatex^®^) and hydroxymethylphosphonium salts (Proban^®^), became commercially available, although with some clear drawbacks. In fact, Proban^®^ is based on the deposition of a tetrakis(hydroxymethyl) phosphonium–urea condensate on the fabric and its successive crosslinking with ammonia. As a consequence, since the flame retardant is just retained within the fabric interstices, formaldehyde may be released during the textile service [19]. Conversely, Pyrovatex^®^ employs a standard pad-dry-cure process covalently linking the flame retardant with the hydroxyls of the cellulosic substrate by means of a methylolated compound: nonetheless, during the first laundry occasion, around 50% of unreacted flame retardant comes off from the fabric [22].

Targeting a “greener” approach, it must be considered that the replacement of already available FRs with equivalent products showing low environmental impact and toxicity must fulfil several requirements. First of all, the application of the new products should be at least as easy as that of the flame retardant being replaced; then, formaldehyde should not be released during the application of the FR onto the fabric or even during service. Moreover, it is important that the new product does not modify the overall features of the treated textile, namely soft touch (i.e., the “hand”), durability, air permeability, dyeability, aesthetics, outward appearance and wearability. Finally, the new flame retardant should exhibit comparable or even reduced costs with respect to the replaced counterpart.

Despite the difficulty of accomplishing this with all these requirements, great efforts have been made in the last 5 to 10 years to assess the suitability of certain biomacromolecules with specific structures and chemical compositions, which may suggest their utilization in the design of flame retarded textiles [23,24,25,26]. Undoubtedly, some proteins, nucleic acids and natural extracts may represent a novel different challenging approach to the fire retardance of fibers and fabrics, also considering that, to date, they have been utilized for different applications, very far from flame retardance. In particular, their uses as edible films, adhesives, food emulsifiers, papermaking, leather finishing systems, and for designing environmental monitoring units and biosensors wearability are well known [27,28,29,30,31]. Several advantages may justify the current interest of the scientific (and industrial) community towards these biomacromolecules as potential new flame retardants: in particular, they show a low environmental impact and can be applied to textile materials by using the already existing industrial finishing plants (i.e., impregnation/exhaustion and spray units). Moreover, some of the selected biomacromolecules (such as whey proteins and caseins) are by-products from the agro-food industry; therefore, their recovery from waste materials and valorization in flame retardant applications may represent a good starting point for avoiding their landfill confinement.

This review work is aimed at describing the main advances and the current limitations about the exploitation of biomacromolecules as effective flame retardants for textiles; in particular, their fire retardance (in terms of resistance to an irradiative heat flux or to a flame spread) is thoroughly correlated with the type of treated substrate (natural or synthetic), the chemical structure and composition of the biomacromolecules, as well as with the achieved final dry add-on.

Finally, some perspectives concerning further developments in the exploitation of the biomacromolecules, such as low environmental impact flame retardants, are presented.

## 2. Whey Proteins as Flame Retardants for Cotton

The first pioneering study [32] dealing with the use of biomacromolecules for conferring flame retardant features in cotton described the potentialities of whey proteins that correspond to approximately 20% of the total amount of proteins in milk (all the rest are made of caseins, as will be discussed later). Whey proteins below 70 °C (i.e., folded or not denatured) show a globular morphology consisting of α-helix structures, with polypeptide chains that include equally allocated acid/basic and hydrophobic/hydrophilic amino acid sequences. α-lactalbumin, β-lactoglobulin, immunoglobulin and bovine serum albumin are the main components of these biomacromolecules. The most characterizing element of whey proteins is sulphur, mainly organized in cysteine and methionine structures that justify the high nutritional values of whey proteins. In addition, these biomacromolecules show high water absorption and solubility, notwithstanding emulsifying and gelatinization capabilities: these specific features justify their wide use for food purposes [33,34,35]. Three main configurations are possible for these biomacromolecules: whey protein isolate (WPI), whey protein hydrolysate (WPH) and whey protein concentrate (WPC).

**Figure 1:**
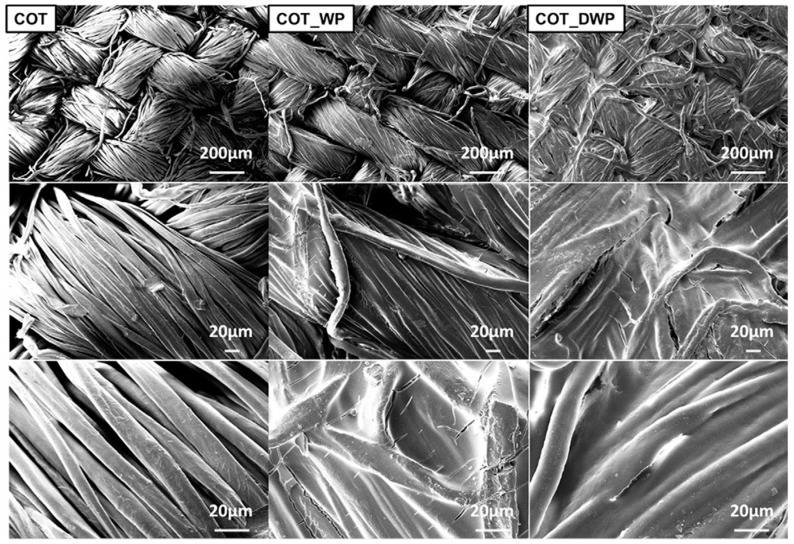
SEM magnifications of cotton (COT), cotton treated with folded (COT_WP) and denatured (COT_DWP) whey proteins. Reproduced with permission from [32]. Copyright 2013, Elsevier.

For fire retardant purposes, cotton fabrics were impregnated with a WPI water suspensions (concentration: 10 wt.%), containing folded (WP) or denatured (DWP) chains, then dried to constant weight in a climatic chamber, achieving a final dry add-on of 25 (for denatured proteins) and 20 wt.% (for not denatured proteins). The typical SEM images obtained before and after the deposition of the protein coatings are shown in Figure 1.

The thermal stability and the flame retardant features was investigated by means of thermogravimetric analyses and flame spread tests in a horizontal configuration. The results from thermogravimetric analyses are compared with untreated cotton in Table 1.

**Figure 2:**
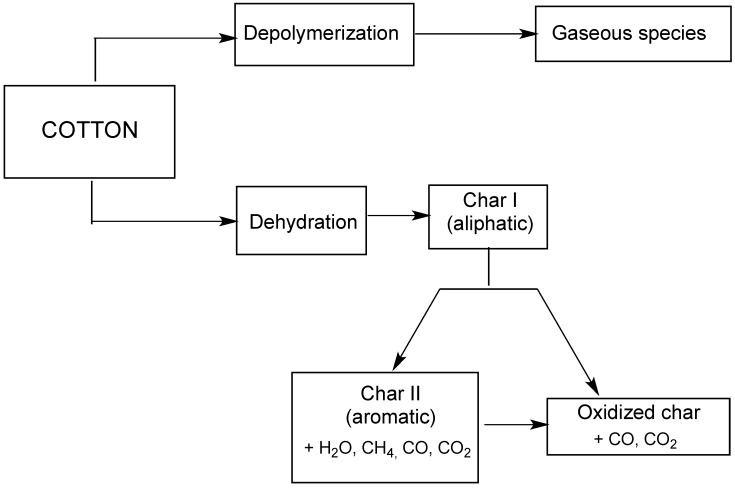
Scheme of cotton degradation.

In nitrogen, cotton degradation proceeds in a single step, with the pyrolysis of cellulose, which, in turn, may involve two competitive paths [14,36], depending on the temperature range (Figure 2); in particular, at low temperatures, glycosyl units decompose, hence giving rise to the formation of char, while at higher temperatures, glycosyl units are likely to depolymerize, favoring the formation of gaseous combustible species. The presence of the whey protein coating, irrespective of the structure of the protein (i.e., folded or denatured), significantly anticipates cotton degradation, as clearly indicated by the T_onset10%_ values; although this seems contradictory, the anticipation of the protein degradation is very important and necessary, as the protein must be activated before the underlying substrate starts decomposing.

In air, cotton typically degrades by three steps. The first (occurring between 300 and 400 °C) includes two competitive paths that trigger the formation of both aliphatic char and gaseous species. Later (from 400 to 800 °C), the aliphatic char is either partially converted into an aromatic (more stable) counterpart, or is oxidized, hence mainly producing CO and CO_2_. Lastly, around 800 °C, the char is further oxidized.

As displayed in Table 1, in air, cotton exhibits two T_max_ decomposition temperatures at 343 and 489 °C. Once again, the decrease of T_onset10%_ values confirms the anticipation caused by the presence of the biomacromolecule coating. On the other hand, a significant increase of the residues at T_max1_ indicates the formation of a quite thermally stable degradation product as a result of the first degradation step. This degradation product is further degraded at higher temperatures, as confirmed by T_max2_ and T_max3_ values; in addition, the final residues are slightly higher than those of untreated cotton.

Table 2 shows the results of the flame spread tests performed in horizontal configuration: overall, the presence of the protein coating is responsible for the decrease of the burning rate and the increase of the residues at the end of the test. Furthermore, the coatings made of not denatured proteins seem more effective in protecting the underlying cellulosic substrate; this finding was ascribed to the better coverage of the fabric provided by the unfolded proteins, which are responsible for the formation of more compact and coherent residues as compared to denatured coatings.

## 3. Caseins as Flame Retardants for Cotton, Polyester and Cotton-Polyester Blends

As mentioned in the previous paragraph, caseins represent the most abundant fraction (around 80%) of milk proteins and are considered co-products during the production of skim milk. αS1-, αS2-, β-, and κ-caseins are the main types of caseins. They differ as far as the structure and phosphorus content are concerned. In particular, κ-caseins include a very limited number of phosphate groups, located in the C-terminal region of the protein; conversely, β-caseins exhibit an entirely phosphorylated structure containing five phosphate groups/mol; αS2-caseins show four different phosphorylated isoforms with a high content of phosphate groups (from 10 to 13 groups/mol). Lastly, αS1-caseins are the most abundant in bovine milk and include eight to nine bound phosphate groups/mol.

Some general uses of these proteins include cheese farming (main usage), whipping, emulsifying, water binding and thickening, notwithstanding their utilization in coatings for papermaking, printing, finishing of synthetic fibers and leather [37].

The flame retardant properties of these proteins were demonstrated on cotton (COT), polyester (PET) and cotton/polyester-rich (COT-PET; polyester content: 65 wt.%) blend fabrics, by employing a procedure similar to that adopted for whey proteins [38,39]. After drying, the final add-on was 20 wt.%.

The results obtained from thermogravimetric analyses (performed both in air and inert atmosphere) are collected in Table 3.

**Figure 3:**
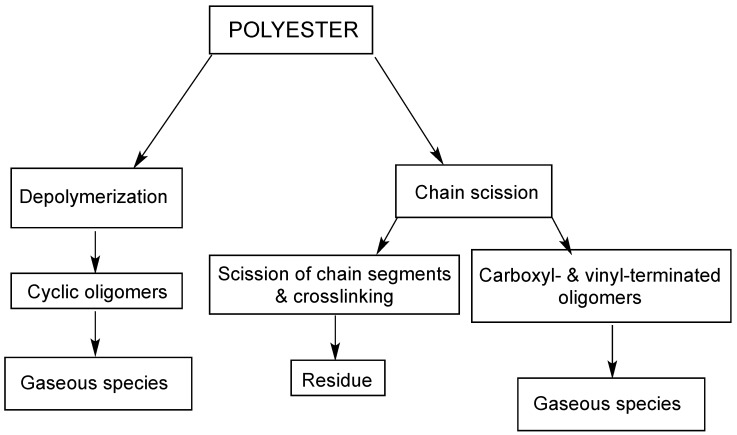
Competitive pathways involved in the thermal and thermo-oxidative degradation of polyester.

As far as polyester is concerned, its degradation in nitrogen takes place according to a single step; 426 °C is the temperature corresponding to the maximum weight loss. In particular, degradation involves heterolytic cleavage reactions or homolytic scissions of ester bonds, which put the char formation in competition with the production of volatile combustible species (Figure 3). In parallel, intramolecular backbiting may promote chain depolymerization, with the formation of carboxyl- and vinyl-terminated oligomers, from which carbon monoxide, carbon dioxide, methane, ethane, formaldehyde, acetaldehyde, benzene and benzaldehyde may originate.

Differently, COT-PET blends degrade according to two autonomous steps, each corresponding to the degradation of the single components of the blend: the temperatures corresponding to the maximum weight loss are 351 and 423 °C for cotton and polyester, respectively. Irrespective of the treated fabric, the presence of the caseins coatings significantly anticipates textile degradation, as shown by the decreased T_onset10%_ values; this finding can be ascribed to the thermal degradation of the phosphate groups positioned on the shell of the protein micelles, which show a catalytic effect on both COT and PET degradation, favoring the development of a stable char (as also revealed by the high residues at the end of the tests), rather than the formation of volatile species.

In air, PET degradation undergoes a two-step pathway. In fact, the two maxima weight losses, located at around 422 and 547 °C appear; they refer to simultaneous β CH-transfer reactions and chain depolymerizations. Conversely, the blends degrade according to a three-step process, showing maxima weight losses at 335, 416 and 525 °C.

The results from the flammability tests (namely, horizontal flame spread tests and Limiting Oxygen Index – LOI – measurements) are collected in Table 4; the presence of the caseins coating, irrespective of the type of fabric substrate, appreciably decreases the burning rate and increases the total burning time, leading to the formation of a very stable char, as revealed by the increased residues. Moreover, all the treated fabrics achieve self-extinction, even after several flame applications. The only “drawback” of the protein coatings relates to PET fabrics, for which the melt dripping phenomenon cannot be suppressed, although the flame stops propagating within 30 mm. Finally, the LOI values significantly increase for cotton (+6% with respect to the untreated fabric) and polyester (+5% with respect to the untreated fabric).

In order to complete the characterization of their fire behavior, the different fabrics (before and after the finishing with caseins) were tested under the cone calorimeter using an irradiative heat flux of 35 kW/m^2^. Time to ignition (TTI), peak of Heat Release Rate (pkHRR) and final residue are shown in Table 5.

First, in accordance with the results of the thermogravimetric analyses, the protein coatings anticipate the ignition of the fabric specimens, but, at the same time, are capable of decreasing the pkHRR values of cotton (−19%), polyester (−2.7%) and cotton-polyester blends (−15%). In addition, the char-forming character of the designed systems coated on polyester or cotton-polyester blends was witnessed by the increased residue at the end of the test.

## 4. Hydrophobins as Flame Retardants for Cotton

Hydrophobins are amphipathic proteins with low molar masses (usually between 7 and 9 kDa) produced by Filamentous fungi [40]. According to the distribution of cysteine and the clustering of hydrophilic and hydrophobic amino acid residues, hydrophobins can be classified as HFBI (i.e., class I) and HFBII (i.e., class II); the latter are highly soluble in aqueous media, giving rise to the formation of soluble hydrophilic aggregates. Conversely, HFBI cannot be dissolved in aqueous media, where they from hydrophobic aggregates [37].

The chemical structure of these proteins shows eight cysteine residues originating from four non-sequential disulphide bonds that stabilize the tertiary structure of hydrophobins; Moreover, they are capable of self-assembling amphipathic monolayers at the hydrophilic–hydrophobic interfaces, thus exhibiting surfactant-like features. Their traditional applications are in the field of surface modifiers, protective coatings and adhesives [27], notwithstanding their uses as emulsifiers, nanoencapsulating and foaming systems in the food industry and as biosensors [28].

**Figure 4:**
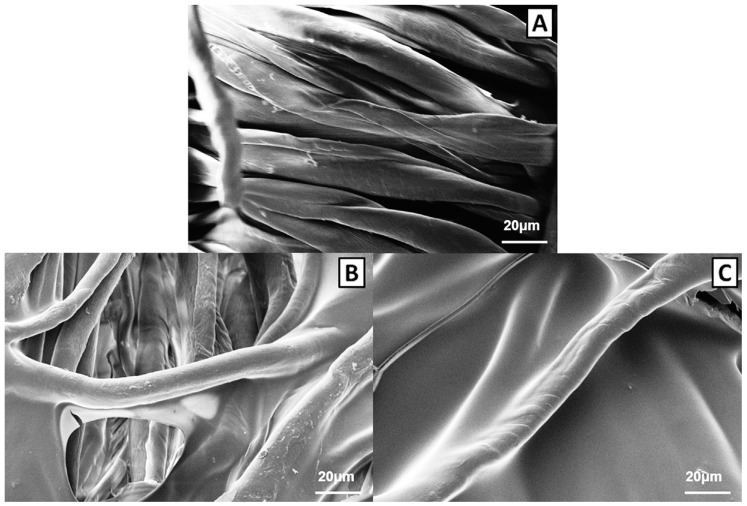
SEM magnifications of untreated cotton (**A**), cotton treated with caseins (**B**) and cotton treated with hydrophobins (**C**). Reproduced with permission from [38]. Copyright 2014, Elsevier.

Their utilization for fire retardant purposes is quite recent; in this context, 5 wt.% aqueous hydrophobin solutions were employed for treating cotton fabrics by means of impregnation; the final dry add-on was around 20 wt.% [38]. Some typical SEM images of cotton before and after the treatment with caseins or hydrophobins are shown in Figure 4.

The results from the thermogravimetric analyses are shown in Table 6. Similarly to the previously discussed biomacromolecules, in nitrogen, the presence of the protein anticipates the degradation of the cellulosic substrate. In fact, T_onset10%_ values decrease for the treated fabrics. At variance, the biomacromolecule coating does not affect T_max1_ values but remarkably increase the final residue, hence confirming its char-forming character.

These findings can also be drawn in air, where the degradation of the treated cotton involves a three-step process: the thermally stable product formed during the first degradation step (which is slightly anticipated—see T_max1_ values in Table 6—in the presence of the protein coating) is further decomposed at higher temperatures (i.e., at T_max2_ and T_max3_), leaving a final residue at 600 °C, marginally higher as compared to the untreated substrate.

The flame spread tests carried out in the horizontal configuration (Table 7) indicate that the hydrophobin coating is able to protect the underlying fabric. In particular, the total burning time increases (+44% with respect to untreated cotton), while the total burning rate decreases (−13%); furthermore, a coherent residue is found at the end of the test. SEM analyses performed on this latter show the formation of unblown bubbles, hence indicating the intumescent-like character of the protein coating due to the cleavage of the disulphide bonds and to the crosslinking of amide groups [27].

Finally, it is noteworthy that in forced-combustion tests, a two-step process occurs when the treated fabrics are exposed to 35 kW/m^2^ heat flux. More specifically, apart from an anticipation of Time to Ignition (−44%), the protein coating remarkably lowers the pkHRR of the first combustion step (−45%); then, during the second combustion step, some cracks develop on the irradiated surface, hence producing some preferential channels that further speed up the process.

## 5. Deoxyribonucleic Acid (DNA) as Flame Retardant for Cotton

**Figure 5:**
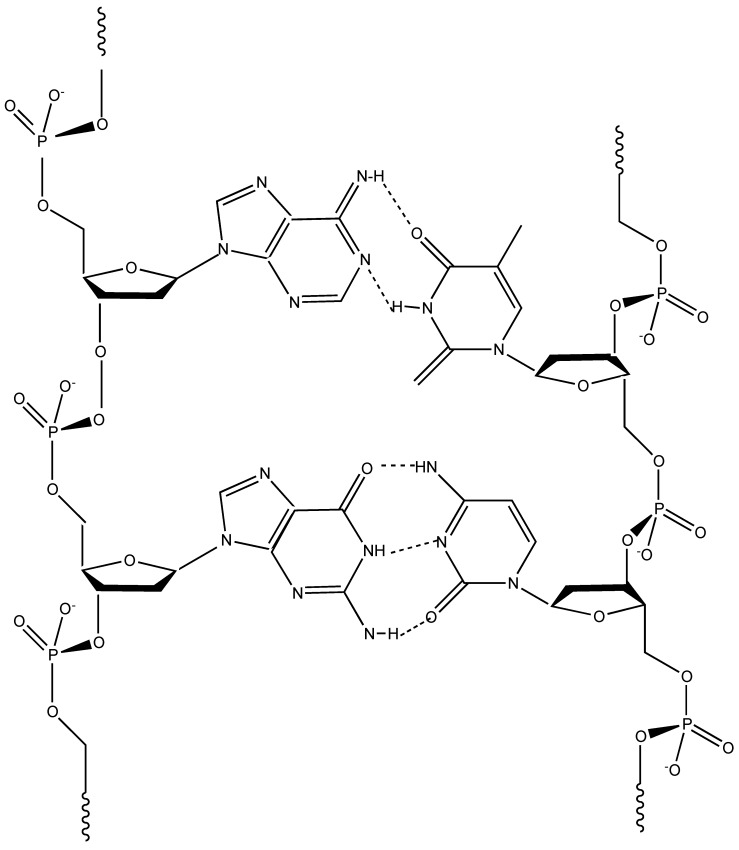
Structure of deoxyribonucleic acids.

DNA (Figure 5) is perhaps one of the most well-known biomacromolecules; it consists of a double helix comprising two long polymer chains of nitrogen-containing bases (namely, adenine, cytosine, guanine and thymine) with backbones made of five-carbon sugars (so-called deoxyribose units) and of phosphate groups connected by ester links. The resulting double helix exploits the H-bonds between the bases that are located side by side and specifically combined (in particular, adenine bases are paired with thymine bases, while cytosine bases with guanine).

One of the main advantages of this particular structure and morphology is that phosphate groups and deoxyribose units are oriented towards the outside of the biomacromolecule, hence being very easily accessible, even in the presence of a flame or an irradiative heat flux.

Among the traditional uses, this biomacromolecule is being employed for fabricating several DNA-based nanomaterials; among them, it is worth mentioning DNA-functionalized carbon nanotubes, DNA-directed nanowires and DNA-linked metal nanoparticles [30]. In addition, it has been utilized for environmental monitoring, designing drugs, for the production of industrial microorganisms and for bio-based sensors [31].

The structure and chemical composition of deoxyribonucleic acid are very intriguing as far as flame retardance is considered as this biomacromolecule shows an intumescent-like behavior [41,42]. In fact, it includes i) deoxyribose units that act as carbon source and blowing agents, ii) bases containing nitrogen for the release of ammonia and iii) phosphate groups that, upon the application of a flame or an irradiative heat source, degrade to phosphoric acid, hence promoting the dehydration of the underlying fabric and the subsequent formation of a stable protective char. Therefore, the heat and mass transfer phenomena taking place between flame and burning fabric are limited by the formed multicellular swollen carbonaceous structure, which acts as an effective physical barrier and is even able to stop the combustion reaction (i.e., extinguishing the flame).

The first pioneering article dates back to 2013 and deals with a commercially available DNA extracted from herring sperm and applied to cotton [43]. As for the proteins described in the previous paragraphs, the DNA powder was dispersed in water (2.5 wt.% concentration) and used for impregnating the cellulosic fabric, reaching a final dry add-on of 19 wt.%. This latter allowed the achievement of self-extinction in horizontal flame spread tests; after the flame application, the treated fabric started to burn very slowly and the flame out was achieved after just 2 s from the ignition; moreover, the sample did not ignite again, even after trying to repeatedly apply the flame. This behavior was further supported by Limiting Oxygen Index tests for which the treated fabric achieved 28% (untreated cotton: 18%) and by forced combustion analyses (for which it was not possible to ignite the treated fabrics using the standard 35 kW/m^2^ irradiative heat flux).

The described fire behavior was attributed to the char-forming ability of DNA, as well as to the dilution effect in the gas phase derived from the decomposition of purine and pyrimidine bases that generate azo-compounds, capable not only od further inducing the char formation, but also of producing non-combustible gases (such as carbon dioxide and nitrogen).

**Figure 6:**
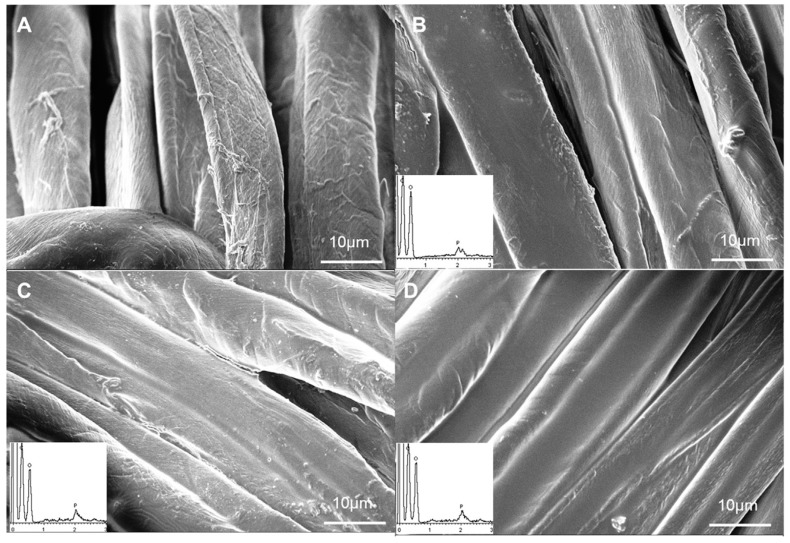
SEM micrographs of COT (**A**), COT_DNA 5% (**B**), COT_DNA 10% (**C**) and COT_DNA 19% (**D**) at 5000× and elemental analyses. Reproduced with permission from [44]. Copyright 2013, Elsevier.

These promising results further stimulated the investigation towards the optimization of the final dry add-on on the treated cotton: in doing so, fabrics with 5, 10 and 19 wt.% add-ons were prepared and thoroughly characterized [44]. Some typical SEM pictures are shown in Figure 6.

Table 8 shows the results of the thermogravimetric analyses performed both in nitrogen and air. Again, independently of the used atmosphere, DNA coatings anticipate the degradation of the cellulosic substrate, as clearly indicated by the drop of T_onset10%_ and T_max1_ values; in addition, this anticipation is strictly dependent on the biomacromolecule content (the lower the add-on, the higher the decomposition onset). As a consequence of the dehydration reactions promoted by the activation of the biomacromolecule, the resulting residue is very stable either in air (beyond 500 °C) or in nitrogen (up to 600 °C) [45].

Table 9 summarizes the results from the flame spread tests performed in the horizontal configuration. Again, flammability is strictly related to the DNA loading on the fabric. More specifically, the lowest add-on (i.e., 5 wt.%) behaves similar to untreated cotton, with the only difference referring to the final residue (12.5% vs. 0%) and the formation of a compact and coherent char that maintains the texture of the pristine fabric. Furthermore, 10 wt.% DNA provides the treated fabrics with self-extinction, although they burn for 35 s before the flame-out occurs. Finally, as previously mentioned, 19 wt.% add-on allows the achievement of self-extinction with the shortest combustion time (only 2 s occurring for the flame out) and the fabric cannot be ignited again.

It is noteworthy that as assessed by SEM-EDX analyses, the intumescent-like behavior of the biomacromolecules was demonstrated by the formation of several small bubbles homogeneously distributed on the burnt fibers and essentially containing carbon, oxygen and phosphorus elements.

These systems were then subjected to forced combustion tests using two different heat fluxes (namely, 35 and 50 kW/m^2^): Table 10 shows the obtained results. From an overall point of view, some outcomes can be summarized as follows:

- at 19 wt.% DNA loading, all the tested specimens did not ignite when irradiated at 35 kW/m^2^.
- at 10 wt.% DNA loading, two out five samples did not ignite when irradiated at 35 kW/m^2^.
- at 5 wt.% DNA loading, all the tested samples ignited when irradiated at 35 kW/m^2^.
- below 19 wt.% DNA loading, the presence of the biomacromolecule was responsible for the anticipation of the ignition (i.e., for the decrease of TTI values); moreover, for all the tested formulations, DNA promoted the reduction of pkHRR values by 50% as a minimum and significantly increased the residues at the end of the tests.

Therefore, in conclusion, it is worth underlining that the overall fire performance of the DNA-treated fabrics was strictly related to the biomacromolecule loading. Indeed, this is responsible for the formation of a continuous and homogeneous coating that covers each single fiber and the fabric interstices; this condition was satisfied only in the presence of the highest nucleic acid add-ons (i.e., 10 and 19 wt.%).

Pursuing the research on this biomacromolecule, the impregnation of cotton was replaced with the layer-by-layer (LbL) technique [46,47,48], coupling DNA with chitosan in a bi-layered assembly [49].

**Figure 7:**
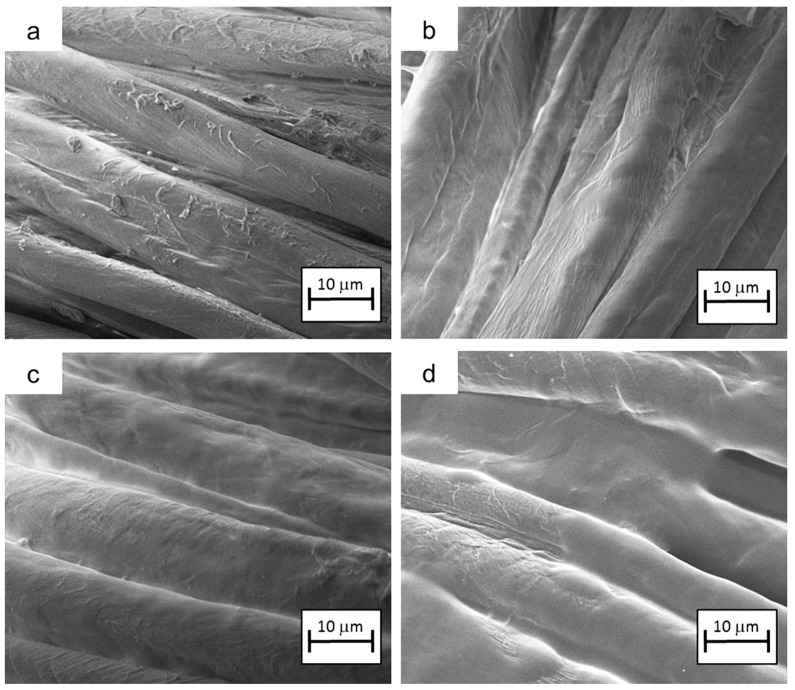
SEM micrographs of untreated cotton (**a**) and fabrics coated with 5 (**b**), 10 (**c**) and 20 (**d**) BL. Reproduced with permission from [49]. Copyright 2013, Elsevier.

In particular, three different numbers of bi-layers (BL), namely 5, 10 and 20, were assembled on the cellulosic substrate; the corresponding dry add-ons were 5, 7 and 15 wt.%, respectively. Some typical SEM images are shown in Figure 7. The results from the flame spread tests performed in horizontal configuration are shown in Table 11, together with the limiting oxygen index values.

First, it is noteworthy that the number of bi-layers the deposited LbL architectures are made of significantly affects the flammability of cotton. In particular, 5 BL is not enough to influence the total burning time or rate, notwithstanding that this assembly is able to increase the final residue (8%). At variance, 10 BL increases the total burning time and lowers the total burning rate, further increasing the residue at the end of the test (48%). Self-extinction is achieved only with the highest number of deposited bi-layers (i.e., 20 BL), which also show the highest LOI values (24%).

Then, the treated fabrics, together with untreated cotton were exposed to an irradiative heat flux of 35 kW/m^2^. The results are summarized in Table 12.

Once again, the presence of the LbL coatings decreases the TTI values, as, upon heating, the biomacromolecule activates, releasing phosphoric acid and starting the dehydration of the underlying textile. Moreover, increasing the number of deposited bi-layers significantly lowers the pkHRR values (up to −40% decrease for 20 BL assemblies). Finally, the forced-combustion behavior of these LbL DNA/chitosan assemblies was compared with that of the bilayers, where DNA was replaced with ammonium polyphosphate, a standard well-known intumescent flame retardant. It was found that using DNA as a component of the assemblies, the phosphorus-nitrogen synergism occurs in a better way than in ammonium polyphosphate/chitosan architectures [50,51].

## 6. Phytic acid (PA) as Flame Retardant for Different Fabrics

**Figure 8:**
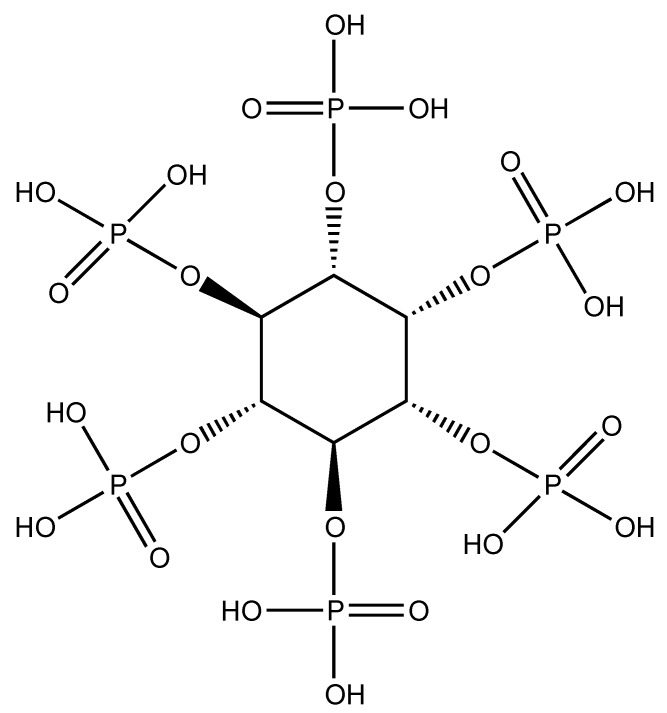
Structure of phytic acid.

Phytic acid (Figure 8) is chemically identified as inositol hexakisphosphate acid; it contains 28 wt.% of phosphorus and it is quite abundant and easily extracted from plants tissues, such as oil seeds, beans and cereal grains, among others [52]. Because of its biocompatibility, non-toxicity and easy recovery, it is being widely employed in the formulation of biosensors, antioxidants, anticancer formulations and cation exchange systems [53,54].

Because of the high content of phosphorus PA, some phytates have been investigated as possible low environmental impact flame retardants for different fabrics. One of the pioneering studies dealing with the use of this bio-based molecule in flame retardance dates back to 2016 and describes the effects on wool fabrics [55]. More specifically, the fabrics were dipped in PA water solutions (concentration ranging from 10 to 200% owf phytic acid), in acidic conditions (pH = 1.2), and then thermally treated at 90 °C for 1 h. Thus, it was possible to obtain different final dry add-ons (i.e., 10.6, 15.0 and 17.9 wt.%).

Table 13 shows the results from the thermogravimetric analyses carried out either in nitrogen or in air.

Overall, the presence of phytic acid significantly increases the thermal stability of wool, irrespective of the used atmosphere, as clearly indicated by the increase of T_onset20%_ and T_onset50%_ values. Furthermore, the char-forming character of PA was witnessed by the increase of the residues at the end of the thermogravimetric tests.

The results of the microscale combustion calorimetry tests are shown in Table 14. The following outcomes can be pointed out. First, the treated fabrics exhibit decreased pkHRR values with respect to untreated wool; furthermore, the pkHRR decrease is correlated with increasing phytic acid loadings on the fabrics. A similar behavior is observed for the heat release capacity (HRC) values. Finally, the protection exerted by the biomolecule is further confirmed by the trend of total heat release (THR) values, which remarkably decrease with increasing the phytic acid loading (8.2, 7.4 and 6.7 vs. 14.0 kJ/g for WOOL_PA10.6, WOOL_PA15.0 and WOOL_PA17.9, vs. untreated wool, respectively).

Then, the same flame retardant system was further investigated, coupling phytic acid with titania nanoparticles (average size: 40 nm) in the presence of 1,2,3,4-butanetetracarboxylic acid (BTCA), employed as a cross-linking agent to improve the adhesion of titania on the wool surface, hence enhancing the washing fastness of the treated fabrics [56]. To this aim, an exhaustion-assisted pad-dry-cure method was employed. The proposed treatment provided the wool fabrics with durability, as they were still self-extinguishing (in vertical flame spread tests) after five washing cycles and capable to achieve the B_1_ classification (according to GB/T 17591-2006 standard) even after 30 washing cycles. Moreover, phytic acid and titania nanoparticles showed a joint flame retardant effect, for which the phosphorus element (provided by PA) favored the creation of a stable intumescent char, while titania acted as a physical bridge to strengthen the created char.

**Figure 9:**
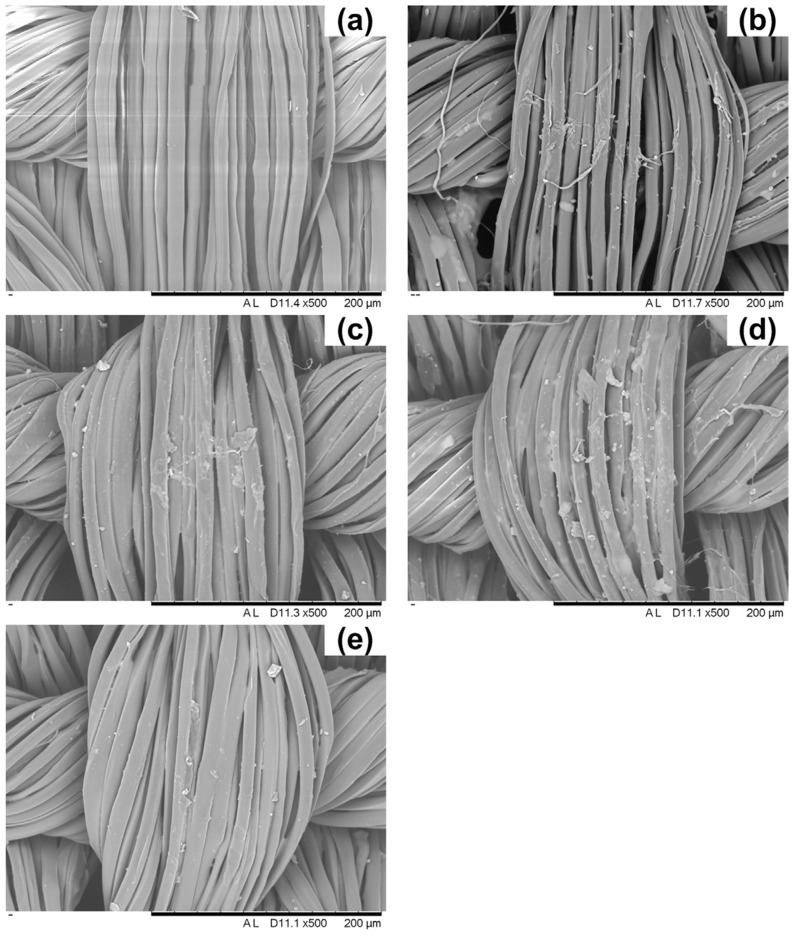
SEM micrographs of the untreated silk fabric (**a**) and the silk fabrics treated with the hybrid sols unmodified (**b**) and modified by APDTMS (**c**), CPTS (**d**) and MPTS (**e**). Reproduced with permission from [57]. Copyright 2018, Elsevier.

Quite recently, phytic acid was exploited in order to design hybrid organic-inorganic flame retardant coatings on silk fabrics [57]. For this purpose, a sol-gel process was employed, using tetraethoxy silane (TEOS) as silica precursor and doping the sol with phytic acid, in the presence of three different coupling agents/cross-linkers, namely 3-aminopropyldimethoxymethylsilane (APTMS), 3-chloropropyltrimethoxysilane (CPTMS) and 3-methacryloxypropyltrimethoxysilane (MPTMS). Thus, it was possible to develop hydrophobic coatings with an enhanced washing fastness on silk fabrics. After modification, the final dry add-on was set at 12.2 wt.%. Some typical SEM picture of silk before and after the sol-gel treatments are shown in Figure 9.

Table 15 shows the results from the thermogravimetric analyses carried out either in nitrogen or in air.

In nitrogen, silk degrades after a two-step process. The first weight loss (below 100 °C) is ascribable to water evaporation; then, the main degradation step (between 260 and 360 °C) originates from the cleavage of peptide bonds, and from the degradation of the side-chain groups in aminoacidic residues [58]. In air, three degradation steps take place: the first two steps are similar to those already described in nitrogen, although occurring at lower temperatures; the last degradation step refers to the partial oxidation of char and of the hydrocarbon species produced in the previous degradation steps to CO and CO_2_.

Irrespective of the chosen atmosphere, the presence of the sol-gel P-doped hybrid coatings causes a decrease of the initial degradation temperature (see T_onset10%_ values) because of the catalytic effect provided by phosphate groups of phytic acid, which form phosphoric and polyphosphoric acids that favor the dehydration of the underlying fabric. Conversely, by comparing T_onset50%_ values, it is noteworthy that the sol-gel coatings are able to exert a good protection on the protein substrate, showing a high char-forming character, as revealed by the increased residues at the end of the tests, apart from the thermal shielding effect derived from the silica ceramic phase.

The results from the microscale combustion calorimetry tests are shown in Table 16: all the main thermal parameters were remarkably reduced in the presence of the different sol-gel coatings, even without the presence of the coupling agents, hence confirming the protection provided by the P-doped silica coating on the underlying fabric.

Finally, the surface roughness provided by the sol-gel treatments ensured high hydrophobic properties of the treated silk with water contact angles above 120°. Moreover, the modification of the sol recipes with the coupling agents enhanced the washing fastness of the treated fabrics, which remained hydrophobic (with water contact angles still above 100°) even after seven laundering cycles. This finding was ascribed to the hydrophobic chains of the selected silane coupling agents, which were strongly interconnected with the polar chain structures of silk.

Recently, wool fabrics were treated with a polyelectrolyte complex made of phytic acid and polyethyleneimine, aiming at obtaining enhanced flame retarded wool fabrics [59]. For this purpose, wool was first dipped in the polyelectrolyte complex solution (pH = 1.5); then, the pH was raised to 4, hence obtaining a water-insoluble coating deposited on the fabric surface. Later, the fabrics were washed in deionized water, pre-dried at 70 °C for 3 min and then cured at 145 °C for 3 min.

Vertical flame spread and LOI tests were carried out for evaluating the reaction to fire of the treated wool fabrics. The results are shown in Table 17. Unlike the untreated fabric, which burned entirely and left a negligible char residue, the coated fabrics show increased performances strictly related to the FR dry add-on: in all cases, the treated wool achieved self-extinction that was retained after 10 washing cycles only in the case of the highest dry add-on (i.e., for WOOL-PA-PEI15 fabrics). This finding was ascribed to the insolubility of the coating on the wool surface, resulting from the interactions of phytic acid and polyethyleneimine, which were also ionically and covalently cross-linked with the fabric substrate.

Table 18 shows the results from microscale combustion calorimetry tests. Once again, the treatment was very effective in lowering both the peak of heat release rate and the total heat release; the observed decrease was much more pronounced as the dry add-on increased. In addition, the char forming character of the designed treatment was witnessed by the remarkable increase of the residues at the end of the tests, hence confirming the protection exerted by the deposited coating on the underlying fabric.

The scientific literature also reports on layer-by-layer treatments for fabrics, where phytic acid was employed as a component of the designed flame retardant assemblies.

The first pioneering study dates back to 2012 and describes the design of LbL assemblies made of anionic phytic acid and cationic chitosan bi-layered assemblies deposited on cotton fabrics [60]. More specifically, 5, 10, 20 and 30 bi-layers were deposited on the cellulosic substrate, changing the pH of aqueous deposition solutions and hence modifying the chemical composition of the final deposited assemblies.

As an example, Table 19 shows the results obtained from the microscale combustion calorimetry, comparing untreated cotton with the fabric coated with 30 bi-layers at pH = 4 (dry add-on: about 16 wt.%). It is clear that the LbL treatment was very effective in lowering the peak of heat release rate (−62%) and the total heat release (−77%), favoring, at the same time, the formation of a stable char, as revealed by the increased residue at the end of the test. Furthermore, the combination of phytic acid and chitosan in the layer-by-layer architecture ensured self-extinction in vertical flame spread tests.

Then, intumescent LbL assemblies consisting of a nitrogen-modified silane hybrid (sol-gel synthesized) and phytic acid were deposited on cotton fabrics. In particular, up to 15 bi-layers were assembled on cotton [61]. Table 20 shows the results of the thermogravimetric analyses performed in nitrogen; in addition to the anticipation of the degradation promoted by the activation of phytic acid layers, these were also responsible for the formation of a stable aromatic char, as confirmed by the increased residues at the end of the tests.

Table 21 shows the results from cone calorimetry data (irradiative heat flux: 35 kW/m^2^). The presence of an increased number of bi-layers determined a significant decrease of the peak of heat release rate and of the total heat release, hence revealing the protection exerted by the deposited assemblies. In addition, the increase of time to ignition was ascribed to the LbL assemblies, which delay the release of volatile combustible species.

Finally, as assessed by the vertical flame spread tests, only 15 bi-layered assemblies were able to provide the underlying cotton with self-extinction.

Recently, a phytic acid layer was deposited between two layers of flexible polysiloxane obtained by means of a sol-gel process, thus giving rise to a tri-layered architecture on polyester fabrics [62]. The vertical flame spread tests indicated that the deposited architecture was capable of preventing melt dripping phenomena, as well as to provide the underlying fabric with self-extinction.

Moreover, cone calorimetry tests showed a significant decrease of the peak of heat release rate (−65%), and total smoke release as well (−72%). Interestingly, the durability of the designed treatment was very high, as after 45 laundering cycles, the treated fabrics did not modify their fire behavior.

## 7. Other Bio-Sourced Products Used as Flame Retardants for Different Fabrics

The recent scientific literature reports some examples dealing with the use of different bio-sourced products (such as natural extracts from vegetables), which show interesting flame retardant properties. The following paragraph summarizes the main recent outcomes.

### 7.1. Banana Pseudostem Sap (BPS)

Banana pseudostem sap (BPS) is recovered by extraction from the pseudostem of the banana tree (*Musa Cavendish*); it contains phosphorous, nitrogen and other metallic constituents [63]).

This natural product was exploited for conferring flame retardant features to cotton [64]. In particular, bleached cotton was first mordanted with 5% tannic acid and 10% alum; afterwards, it was impregnated with banana pseudostem sap water solutions, either non-diluted (1:0) or diluted (1:1 and 1:2), keeping cotton:banana pseudostem sap at 1:10 ratio. Each impregnation was performed for 30 min at alkaline pH. The treated fabrics were then dried at 110 °C for 5 min.

Table 22 collects the results from the vertical flame spread tests: it is worth noting that despite the impossibility of providing the treated fabrics with self-extinction, the proposed treatment appreciably changes the flame retardant behavior of the cellulosic substrate, increasing the total burning time and decreasing the burning rate. This is also demonstrated by the significant increase of limiting oxygen index values found for the treated samples.

### 7.2. Pomegranate Rind Extract (PRE)

This wastage agricultural product contains nitrogen (in different forms, namely: ammonium salt, hexacontanoic acid, nitrogen based carbamic acid, aminoguanidine, hydrazine, ethanamine, 1,3 di amminoguanidine, asparagines and piperidine) and several components (aromatic phenolic groups, inorganic metallic salts, metallic oxides) which can be successfully exploited for conferring flame retardant properties to cellulosic textiles. In particular, the FR efficiency of PRE on jute has recently been assessed [65]. For this purpose, jute fabrics were impregnated for 30 min separately in PRE solutions kept at three different pH values (4.5, 7 and 10); during impregnation, a 1:20 ratio of the fabric to liquor was maintained. Finally, the fabrics were dried at 110 °C for 5 min.

Table 23 collects the results from the vertical flame spread tests. It is noteworthy that the ease of flammability of the treated fabrics is strictly related to the pH of PRE solutions: in particular, alkaline conditions provided the best performances, allowing the achievement of self-extinction and showing the lowest burning rate and LOI values as well.

### 7.3. Tannins

**Figure 10:**
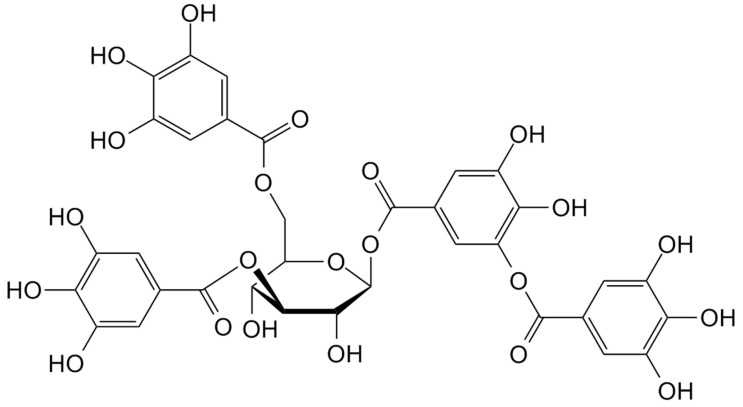
Structure of tannin.

Tannins (Figure 10) are non-toxic, inexpensive and abundant polyphenolic oligomers extracted from biomass. Three types of tannins (i.e., hydrolyzable, complex, and condensed) are available, although condensed tannins denote 90% of the world production. Because of their aromatic structure, tannins possess high resistance to chemicals and high thermal stability; moreover, they show low thermal conductivity [66]. These peculiarities suggest the utilization of these macromolecules for the design of thermal insulating materials and flame retardants [67]. Regarding the latter application, their suitability as effective flame retardants for silk fabrics has recently been demonstrated [68]. In particular, condensed tannin was extracted from *Dioscorea cirrhosa* tuber and utilized for impregnating silk fabrics. The effect of different experimental parameters (namely: pH of the impregnation solutions, temperature and concentration of the extract) was thoroughly evaluated.

The treated silk fabrics showed limiting oxygen index values beyond 27%; the treatments with tannin ensured self-extinction, which was maintained even after 20 laundering cycles (the char length was always below 12 cm). The results from the microscale combustion calorimetry are shown in Table 24: the decrease of all the parameters for the treated fabrics highlights the effectiveness of the proposed treatments, hence suggesting that tannins lower the formation of volatile and flammable pyrolysis products during the combustion process.

Finally, it is worth noting that treatment with tannins, apart from flame retardance, was able to provide silk with antibacterial and antioxidant activities.

### 7.4. Lignin

**Figure 11:**
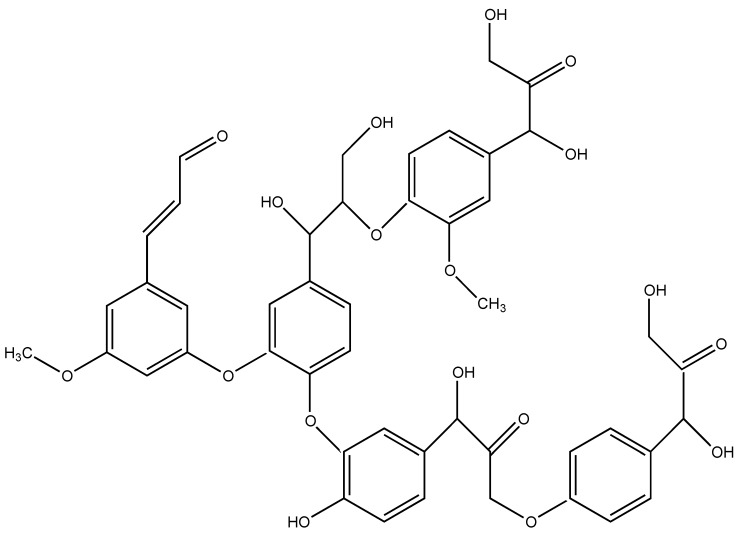
General structure of lignin.

Lignin is the second most abundant natural material after cellulose and is easily extracted from plant cells [69]. Its structure (Figure 11) suggests that this biomacromolecule could act as a potential carbon source when combined with intumescent flame retardant additives in bulky polymers, as it bears phenylpropane units together with aliphatic/aromatic hydroxyls: this peculiarity has been clearly demonstrated in several scientific papers [70,71,72,73]. Lignin and some derivatives have also been exploited for preparing flame retardant fibers (through melt spinning) and subsequently FR fabrics.

Polylactic acid was compounded through melt extrusion with lignin derived from wood waste, in the presence of different amounts of ammonium polyphosphate (APP) [74]. Then, the spinnability of the obtained compounds was assessed. In particular, it was possible to produce flame retarded multifilaments loaded with the intumescent formulation (i.e., lignin + APP) not exceeding 10 wt.% loading. Thermogravimetric analyses carried out in nitrogen showed a slightly improved thermal stability for the compounds containing the two additives; at the same time, the residues at 500 °C increased because of the presence of lignin and its charring capacity. Forced combustion tests demonstrated that the combination of lignin (5 wt.% loading) together with APP (5 wt.% loading) did not increase the time to ignition, but was capable of remarkably lowering the heat release rate of the polymer matrix (about −32%), thanks to the intumescent character of the FR compound, which promoted the formation of a stable aromatic char. Moreover, the same compound showed V0 classification in vertical flame spread tests.

Very recently, polylactic acid was compounded with kraft lignin (used as carbon source) and a commercial phosphorus/nitrogen-based flame retardant containing APP (employed as acidic source), using a melt blending technique [75]. A modified polyester-based plasticizer was also added to the compound in order to assist the spinnability of the resulting FR blends. The melt spinnability of these latter was investigated; in particular, the compounds containing up to 7 wt.% of lignin were spinnable in the presence of 10 wt.% of plasticizer. Finally, the fire behavior of the knitted fabrics produced from multifilament yarns was assessed by forced combustion tests: the combination of the two additives remarkably decreased the heat release rate (−59%) and total heat release (−61%), favoring, at the same time, the formation of a stable residue.

Pursuing this research, the same group succeeded in preparing intumescent flame retarded sheath/core bicomponent melt-spun fibers derived from polylactic acid single polymer composites. For this purpose, a highly crystalline polylactic acid-containing FR was employed for the core component, while an amorphous PLA was used for the sheath component of melt-spun bicomponent fibers [76]. A modified polyester-based plasticizer was also added to the core component in order to facilitate the spinnability of the resulting FR blends. Thus, it was possible to produce thermally bonded non-woven fabric samples from multifilament bicomponent fibers. Forced combustion tests showed a remarkable decrease (−46%) of heat release rate with respect to pure PLA nonwoven counterparts, together with a significant increase (+34%) of the residues at the end of the tests.

## 8. Conclusions and Future Perspectives

Ten years ago, thinking about the use of biomacromolecules or bio-sourced extracts as low environmental impact alternatives to traditional flame retardants was practically impossible. Undoubtedly, the discovery of the FR potentialities of these products has been greatly stimulated by the severe and stringent directives from the EU and the USA regarding the toxicity and in some cases, the carcinogenicity of some of the currently employed flame retardants. Therefore, the search for “green” FR products has produced an increased number of scientific studies that clearly demonstrate the feasibility and suitability of biomacromolecules, especially for flame retarded textiles.

In this context, several experimental parameters have been considered and correlated to the FR performances of these products: temperature, pH and isoelectric point of the aqueous solutions/suspensions, the chemical structure of the biomacromolecules, the involved flame retardant mechanism, the final dry add-on on the fabrics, and the methods employed for the textile FR finishing, among others. By tuning and optimizing these parameters, it was possible to design effective flame retardant systems for different textile substrates.

Despite the great potentialities provided by biomacromolecules or bio-sourced extracts, some challenging issues are currently under debate.

To date, the technology that has been developed for designing textile finishing treatments with FR biomacromolecules is still at a lab-scale level. As a consequence, at present, it is not possible to foresee their potentialities at an industrial (or, at least, pre-industrial) scale. Moreover, the possibility of scaling-up this green know-how is still being evaluated: apart from the flame retardant performances, the cost-effectiveness of the biomacromolecules/bio-sourced extracts represents the key point that will help when taking the final decision. Actually, DNA/nucleic acids, which, among the reviewed systems, seem to show the highest potential for flame retarded textiles, are very expensive, notwithstanding that high purity in not necessary at all [77]. Therefore, the industrial exploitation requires an acceptable reduction of the related supply costs. However, it is expected that the extraction processes of the biomacromolecules and the related technologies will be remarkably enhanced in the next years, hence leading to higher yields and adequate purity levels, specifically suitable for flame retardant purposes.

Moreover, some of the biomacromolecules/bio-sourced extracts show an intrinsic added-value: in fact, they are crops, wastes or by-products derived from the agro-food industry. In this regard, any possible valorization, reducing or even preventing their landfill confinement, is becoming very important, also within the circular economy concept.

As discussed in the review, only a limited number of biomacromolecules/bio-sourced extracts is able to provide the textile substrates with a durable finishing FR treatment. In fact, most of the green FRs are highly soluble in water, hence showing a very limited washing fastness that is conversely very often mandatory in the textile field. Despite this, several attempts have been made to overcome this limitation, always keeping in mind that low environmental impact FRs should require green strategies for being permanently linked to the underlying textile substrate.

A further drawback that somehow limits the use of biomacromolecules as effective flame retardant for fabrics refers to the change in comfort (i.e., “hand” or “soft touch”), which is mostly lost after the FR treatment. In fact, the application of the biomacromolecules at loadings suitable for achieving acceptable flame retardant performances significantly increases the stiffness of the treated textiles, hence making them less wearable and comfortable. Indeed, this is still an open issue which has not found any practical solution yet.

In brief, further advances in the design and development of low environmental impact flame retardant biomacromolecules/bio-sourced extracts can be foreseen for the very near future, paving the path towards increased sustainability.

## Figures and Tables

**Table 1:** Thermogravimetric data of untreated and WP-treated cotton fabrics.

*Atmosphere: N_2_*								
Sample	T_onset10%_ (°C)	T_max1_ * (°C)	T_max2_ * (°C)	T_max3_ * (°C)	Residue @ T_max1_ * (%)	Residue @ T_max2_ * (%)	Residue @ T_max1_ * (%)	Residue @ 600 °C (%)
COT	329	362	-	-	-	-	45.0	8.0
COT_WP	276	355	-	-	-	-	45.0	18.0
COT_DWP	294	366	-	-	-	-	45.5	17.0
*Atmosphere: Air*								
COT	323	343	489	-	48.0	2.0	-	<1.0
COT_WP	283	341	487	580	57.0	14.0	2.5	1.5
COT_DWP	292	345	496	575	56.0	13.0	3.0	2.5

* From dTG curves.

**Table 2:** Horizontal flame spread data for untreated and treated cotton fabrics.

Sample	Total Burning Time (s)	Burning Rate (mm/s)	Final Residue (%)
COT	78	1.5	-
COT_WP	126	1.0	30
COT_DWP	133	1.1	5

**Table 3:** Thermal and thermo-oxidative stability of the untreated and treated fabrics.

*Atmosphere: N_2_*								
Sample	T_onset10%_ (°C)	T_max1_ * (°C)	T_max2_ * (°C)	T_max3_ * (°C)	Residue @ T_max1_ * (%)	Residue @ T_max2_ * (%)	Residue @ T_max3_ * (%)	Residue @ 600 °C (%)
COT	319	354	-	-	41.0	-	-	2.0
COT_Casein	272	337	-	-	49.0	-	-	21.0
PET	400	426	-	-	51.0	-	-	14.0
PET_Casein	315	397	-	-	53.0	-	-	22.0
COT-PET	332	351	423	-	73.0	37.0	-	15.0
COT-PET_Casein	304	334	405	-	75.0	42.0	-	22.0
*Atmosphere: air*								
COT	318	339	478	-	48.0	4.0	-	<1
COT_Casein	242	327	482	-	51.0	10.0	-	<1
PET	392	422	547	-	47.5	1.5	-	0
PET_Casein	310	404	538	-	50.5	13.0	-	2
COT-PET	323	339	419	508	79.0	37.0	7.0	1
COT-PET_Casein	311	335	416	525	82.0	43.0	9.5	2

* From derivative curves.

**Table 4:** Results for untreated and caseins-treated fabrics from horizontal flame spread tests.

Sample	Total Burning Time (s)	Burning Rate (mm/s)	Residue (%)	Dripping	Self-Extinction	LOI (%)
COT	78	1.3	-	No	No	18
COT_Casein	75	0.4	86	No	Yes	24
PET	57	1.8	43	Yes	No	21
PET_Casein	54	0.6	77	Yes	Yes	26
COT-PET	104	1.1	34	No	No	19
COT-PET_Casein	171	0.7	55	No	Yes	21

**Table 5:** Cone calorimetry data for untreated and caseins-treated fabrics.

Sample	TTI (s)	pkHRR * (kW/m^2^)	ΔPHRR (%)	Residue (%)
COT	18	52	-	1
COT_Casein	10	42	−19	3
PET	112	72	-	2
PET_Casein	62	70	−2.7	11
COT-PET	30	60	-	3
COT-PET_Casein	12	51	−15	5

* Experimental error: ±5%.

**Table 6:** Results from thermogravimetric analyses for untreated (COT) and hydrophobin-treated (COT-H) cotton fabrics.

*Atmosphere: N_2_*								
Sample	T_onset10%_ (°C)	T_max1_ * (°C)	T_max2_ * (°C)	T_max3_ * (°C)	Residue @ T_max1_ * (%)	Residue @ T_max2_ * (%)	Residue @ T_max3_ * (%)	Residue @ 600 °C (%)
COT	329	362	-	-	48.0	-	-	8.0
COT_H	295	362	-	-	45.0	-	-	19.0
*Atmosphere: air*								
COT	324	347	492	-	48.0	4.0	-	<1
COT_H	292	336	499	620	61.0	14.0	3.0	4.0

* From derivative curves.

**Table 7:** Results from horizontal flame spread tests for untreated (COT) and hydrophobin-treated (COT-H) cotton fabrics.

Sample	Total Burning Time (s)	Burning Rate (mm/s)	Residue (%)
COT	72	1.5	0
COT_H	104	1.1	19

**Table 8:** Results from thermogravimetric analyses for untreated (COT) and DNA-treated (COT_DNA) cotton fabrics.

*Atmosphere: N_2_*						
Sample	T_onset10%_ (°C)	T_max1_ * (°C)	T_max2_ * (°C)	Residue @ T_max1_ * (%)	Residue @ T_max2_ * (%)	Residue @ 600 °C (%)
COT	335	366	-	46.0	-	8.0
COT_DNA_5%	285	318	-	63.0	-	30.0
COT_DNA_10%	265	314	-	64.0	-	34.0
COT_DNA_19%	243	309	-	67.0	-	35.0
*Atmosphere: air*						
COT	324	347	492	45.0	4.0	0
COT_DNA_5%	282	313	506	65.0	19.0	8.0
COT_DNA_10%	263	302	511	69.0	24.0	13.0
COT_DNA_19%	238	299	515	68.0	29.0	19.0

* From derivative curves.

**Table 9:** Results from horizontal flame spread tests performed on untreated and DNA-treated cotton fabrics.

Sample	Total Burning Time (s)	Char Length (mm)	Burning Rate (mm/s)	Residue (%)	Note
COT	66	100	1.5	0	-
COT_DNA_5%	64	100	1.6	12.5	-
COT_DNA_10%	18	35	1.9	67.0	Flame out for 3/3 specimens
COT_DNA_19%	2	6	3.0	98.0	Flame out for 3/3 specimens

**Table 10:** Cone calorimetry data of untreated and DNA-treated cotton fabrics.

Sample	TTI (s)	pkHRR (kW/m^2^)	ΔpkHRR (%)	Residue (%)	Note
*Heat Flux: 35 kW/m^2^*					
COT	45	125	-	<3	
COT_DNA_19%	No ignition			24	5/5 samples do not ignite
COT_DNA_10%	19	62	-50	15	2/5 samples do not ignite
COT_DNA_5%	24	68	-56	15	
*Heat Flux: 50 kW/m^2^*					
COT	16	128	-	<3	
COT_DNA_19%	10	51	-60	17	

**Table 11:** Flammability data of untreated and LbL-treated cotton fabrics.

Sample	Total Burning Time (s)	Total Burning Rate (mm/s)	Residue (%)	Note	LOI (%)
COT	80	1.5	-	-	18
COT_5BL	78	1.5	8	-	21
COT_10BL	125	1.2	48	-	23
COT_20BL	30	1.0	88	Flame out for 3/3 specimens	24

**Table 12:** Cone calorimetry data of untreated and LbL-treated cotton fabrics.

Sample	TTI (s)	pkHRR (kW/m^2^)	Residue (%)
COT	39	97	2
COT_5BL	17	73	11
COT_10BL	20	60	12
COT_20BL	23	57	13

**Table 13:** Results from the thermogravimetric analyses for untreated (WOOL) and PA-treated (WOOL_PA) wool fabrics.

*Atmosphere: N_2_*			
Sample	T_onset20%_ (°C)	T_onset50%_ (°C)	Residue @ 700 °C (%)
WOOL	265	344	22.3
WOOL_PA10.6	271	358	33.2
WOOL_PA15.0	275	386	37.6
WOOL_PA17.9	272	426	38.0
*Atmosphere: Air*			
WOOL	270	400	2.8
WOOL_PA10.6	278	454	26.1
WOOL_PA15.0	278	460	31.5
WOOL_PA17.9	280	478	36.0

**Table 14:** Results from the microscale combustion calorimetry tests for untreated (WOOL) and PA-treated (WOOL_PA) wool fabrics.

Sample	HRC (J/g·K)	pkHRR (W/g)	THR (kJ/g)
WOOL	130	132	14.0
WOOL_PA10.6	85	90	8.2
WOOL_PA15.0	78	81	7.4
WOOL_PA17.9	74	77	6.7

**Table 15:** Results from the thermogravimetric analyses for untreated and sol-gel-treated silk fabrics.

*Atmosphere: N_2_*			
Sample	T_onset10%_ (°C)	T_onset50%_ (°C)	Residue @ 700 °C (%)
SILK	286	388	31.1
SILK+Unmodified sol	260	467	44.2
SILK+APTMS-modified sol	283	563	48.2
SILK+CPTMS-modified sol	280	557	48.7
SILK+MPTMS-modified sol	275	520	47.1
*Atmosphere: Air*			
SILK	278	370	0.3
SILK+Unmodified sol	268	464	18.2
SILK+APTMS-modified sol	276	456	24.1
SILK+CPTMS-modified sol	248	450	23.8
SILK+MPTMS-modified sol	255	474	24.4

**Table 16:** Results from microscale combustion calorimetry for untreated and sol-gel treated silk fabrics.

Sample	HRC (J/g·K)	pkHRR (W/g)	THR (kJ/g)
SILK	144	145	9.0
SILK+Unmodified sol	75	75	5.8
SILK+APTMS-modified sol	65	66	5.2
SILK+CPTMS-modified sol	67	67	4.8
SILK+MPTMS-modified sol	71	72	4.9

**Table 17:** Results from vertical flame spread and LOI tests for wool before and after the treatments with the polyelectrolyte complex.

Sample	Dry Add-On (wt.%)	Char Length (cm)	Self-Extinction	LOI (%)
WOOL	-	30	NO	23.6
WOOL-PA-PEI5	12.2	9.1	YES	31.8
WOOL-PA-PEI10	20.2	8.2	YES	33.3
WOOL-PA-PEI15	26.0	7.8	YES	36.8

**Table 18:** Results from microscale combustion calorimetry for wool before and after the treatments with the polyelectrolyte complex.

Sample	pkHRR (W/g)	THR (kJ/g)	Char Residue (%)
WOOL	139	13.3	17.3
WOOL-PA-PEI5	100	8.5	28.6
WOOL-PA-PEI10	90	7.9	30.5
WOOL-PA-PEI15	84	7.4	32.4

**Table 19:** Results from microscale combustion calorimetry for untreated cotton and cotton treated with 30 bi-layers at pH = 4.

Sample	pkHRR (W/g)	THR (kJ/g)	Char Residue (%)
COT	259	12.0	5.6
COT30BLpH4	99	2.8	41.7

**Table 20:** Results from the thermogravimetric analyses carried out in inert atmosphere.

Sample	T_5%_ (°C)	T_max1_ (°C)	Residue @700 °C (%)
COT	310	375	4.6
COT-5BL	277	343	31.0
COT-10BL	286	333	36.1
COT-15BL	242	312	39.9

**Table 21:** Cone calorimetry data of the untreated and LbL-treated cotton fabrics.

Sample	TTI (s)	pkHRR (kW/m^2^)	THR (kW/m^2^)	Residue (%)
COT	26	186	10.0	8.7
COT_5BL	40	145	7.3	28.0
COT_10BL	61	138	7.6	30.5
COT_20BL	77	128	6.3	36.4

**Table 22:** Vertical flame spread data for cotton fabrics before and after treatment with different solutions of banana pseudostem sap.

Sample	Add-On (wt.%)	Total Burning Time (s) (Flame Time + Afterglow Time)	Total Burning Rate (mm/min)	LOI (%)
COT-Mordanted	-	60 + 0	250	18
COT_BPS1:2	2.0	10 + 500	29.4	26
COT_BPS1:1	3.5	7 + 680	21.8	28
COT_BPS1:0	4.5	4 + 900	16.6	30

**Table 23:** Vertical flame spread data for jute fabrics before and after treatment with pomegranate rind extract solutions at different pH values.

Sample	Add-on (wt.%)	Total Burning Time (s) (Flame Time + Afterglow Time)	Total Burning Rate (mm/min)	LOI (%)
JUTE	-	100 + 80	1.38	22
JUTE_PRE-pH4.5	6.2	0 + 1560	0.16	33
JUTE_PRE-pH7	6.8	0 + 2400	0.10	35
JUTE_PRE-pH10	7.5	0 + 600 *	0.08	38

* self-extinction achieved within 60 mm char length.

**Table 24:** Results from microscale combustion calorimetry for silk before and after the treatment with tannin.

Sample	pkHRR (W/g)	THR (kJ/g)	HRC (J/g·K)
SILK	134	8.6	138
SILK treated with 37.5 g/L extract	123	8.2	119
SILK treated with 300 g/L extract	114	7.5	111
